# On Benchmarking for Concurrent Runtime Verification

**DOI:** 10.1007/978-3-030-71500-7_1

**Published:** 2021-02-24

**Authors:** Luca Aceto, Duncan Paul Attard, Adrian Francalanza, Anna Ingólfsdóttir

**Affiliations:** 8 Universidad Autónoma de Madrid, Madrid, Spain; 9 University of Twente, Enschede, The Netherlands; 10 University of Malta, Msida, Malta; 11 Reykjavík University, Reykjavík, Iceland; 12 Gran Sasso Science Institute, L’Aquila, Italy

**Keywords:** Runtime verification, Synthetic benchmarking, Software performance evaluation, Concurrent systems

## Abstract

We present a synthetic benchmarking framework that targets the systematic evaluation of RV tools for message-based concurrent systems. Our tool can emulate various load profiles via configuration. It provides a multi-faceted view of measurements that is conducive to a comprehensive assessment of the overhead induced by runtime monitoring. The tool is able to generate significant loads to reveal edge case behaviour that may only emerge when the monitoring system is pushed to its limit. We evaluate our framework in two ways. First, we conduct sanity checks to assess the precision of the measurement mechanisms used, the repeatability of the results obtained, and the veracity of the behaviour emulated by our synthetic benchmark. We then showcase the utility of the features offered by our tool in a two-part RV case study.

### 1 Introduction

Large-scale software design has shifted from the classic monolithic architecture to one where applications are structured in terms of independently-executing asynchronous components [17]. This shift poses new challenges to the validation of such systems. Runtime Verification (RV) [9,27] is a *post-deployment* technique that is used to complement other methods such as testing [46] to assess the *functional* (*e.g.* correctness) and *non-functional* (*e.g.* quality of service) aspects of concurrent software. RV relies on instrumenting the system to be analysed with monitors, which inevitably introduce *runtime overhead* that should be kept minimal [9]. While the worst-case complexity bounds for monitor-induced overheads can be calculated via standard methods (see, *e.g.* [40,14,1,28]), *benchmarking* is, by far, the preferred method for assessing these overheads [9,27]. One reason for

* Supported by the doctoral student grant (No: 207055-051) and the TheoFoMon project (No: 163406-051) under the Icelandic Research Fund, the BehAPI project funded by the EU H2020 RISE under the Marie Skłodowska-Curie action (No: 778233), the ENDEAVOUR Scholarship Scheme (Group B, national funds), and the MIUR project PRIN 2017FTXR7S IT MATTERS.

this choice is that benchmarks tend to be more *representative* of the overhead observed in practice [30,15]. Benchmarks also provide a *common platform* for gauging workloads, making it possible to *compare* different RV tool implementations, or rerun experiments to *reproduce* and *confirm* existing results.

The utility of a benchmarking tool typically rests on two aspects: *(i)* the *coverage* of scenarios of interest, and *(ii)* the quality of *runtime metrics* collected by the benchmark harness. To represent scenarios of interest, benchmarking tools generally employ suites of third-party *off-the-shelf (OTS) programs* (*e.g.* [60,11,59]). OTS software is appealing because it is readily usable and inherently provides realistic scenarios. By and large, benchmarks rely on a range of OTS programs to broaden the coverage of real-world scenarios (*e.g.* DaCapo [11] uses 11 open-source libraries). Yet, using OTS programs as benchmarks poses challenges. By design, these programs do *not* expose hooks that enable harnesses to easily and accurately gather the runtime metrics of interest. When OTS software is treated as a black box, benchmarks become harder to control, impacting their ability to produce repeatable results. OTS software-based benchmarks are also limited when inducing specific edge cases—this aspect is critical when assessing the safety of software, such as runtime monitors, that are often assumed to be *dependable*. Custom-built *synthetic programs* (*e.g.* [35]) are an alternative way to perform benchmarking. These tend to be less popular due to the perceived drawbacks associated with developing such programs from scratch, and the lack of 'real-world' behaviour intrinsic to benchmarks based on OTS software. However, synthetic benchmarks offer benefits that offset these drawbacks. For example, *specialised* hooks can be built into the synthetic set-up to collect a broad range of runtime metrics. Moreover, synthetic benchmarks can also be *parametrised* to emulate variations on the same core benchmark behaviour; this is usually harder to achieve via OTS programs that implement narrow use cases.

Established benchmarking tools such as SPECjvm2008 [60], DaCapo [11], ScalaBench [59] and Savina [35]—developed for the JVM—feature extensively in the RV literature, *e.g.* see [48,19,18,54,13,45]. Apart from [45], these works assess the runtime overhead solely in terms of the *execution slowdown*, *i.e.,* the difference in running time between the system fitted with and without monitors. Recently, the International RV competition (CRV) [8] advocated for other metrics, such as *memory consumption*, to give a more qualitative view of runtime overhead. We hold that RV set-ups that target concurrency benefit from other facets of runtime behaviour, such as the *response time*, that captures the overhead between communicating components. Tangibly, this metric reflects the *perceived reactiveness* from an end-user standpoint (*e.g.* interactive apps) [50,61,58,21]; more generally, it describes the *service degradation* that must be accounted for to ensure adequate quality of service [15,39]. Arguably, benchmarking tools like the ones above (*e.g.* Savina) should provide even more. Often, RV set-ups for concurrent systems *need* to scale in response to dynamic changes, and the capacity for a benchmark to emulate *high loads* cannot be overstated. In actual fact, these loads are known to assume characteristic *profiles* (*e.g.* spikes or uniform rates), which are hard to administer with the benchmarks mentioned earlier.

The state of the art in benchmarking for concurrent RV suffers from another issue. Existing benchmarks—conceived for validating other tools—are repurposed for RV and often *fail* to cater for concurrent scenarios where RV is realistically put to use. SPECjvm2008, DaCapo, and ScalaBench lack workloads that leverage the JVM concurrency primitives [52]; meanwhile, [12] shows that the Savina microbenchmarks are essentially sequential, and that the rest of the programs in the suite are sufficiently simple to be regarded as microbenchmarks too. The CRV suite mostly targets *monolithic* software with limited concurrency, where the potential for scaling up to high loads is, therefore, severely curbed.

This paper presents a benchmarking framework for evaluating *runtime monitoring* tools written for verification purposes. Our tool focusses on component systems for asynchronous message-passing concurrency. It generates synthetic system models following the *master-slave* architecture [61]. The master-slave architecture is pervasive in distributed (*e.g.* DNS, IoT) and concurrent (*e.g.* web servers, thread pools) systems [61,29], and lies at the core of the MapReduce model [22] supported by Big Data frameworks such as Hadoop [63]. This justifies our aim to build a benchmarking tool targeting this architecture. Concretely:

– We detail the design of a *configurable benchmark* that emulates various master-slave models under commonly-observed load profiles, and gathers different metrics that give a *multi-faceted* view of runtime overhead, Sec. 2.
– We demonstrate that our synthetic benchmarks can be engineered to approximate the *realistic behaviour* of web server traffic with high degrees of precision and repeatability, Sec. 3.1.
– We present a case study that *(i)* shows how the load profiles and parametrisability of our benchmarks can produce edge cases that can be measured through our performance metrics to asses runtime monitoring tools in a *comprehensive* manner, and *(ii)* confirms that the results from *(i) coincide* with those obtained via a real-world use case using OTS software, Sec. 3.2.

### 2 Benchmark Design and Implementation

Our set-up can emulate a range of system models and subject them to various load types. We consider master-slave architectures, where one central process, called the *master*, creates and allocates tasks to *slave* processes [61]. Slaves work concurrently on tasks, relaying the result to the master when ready; the latter then combines these results to yield the final output. Our slaves are an *abstraction* of sets of cooperating processes that can be treated as a single unit.

#### 2.1 Approach

We target concurrent applications that execute on a single node. Nevertheless, our design adheres to three criteria that facilitate its extension to a distributed setting. Specifically, components: *(i)* share neither a common clock, *(ii)* nor memory, and *(iii)* communicate via asynchronous messages. Our present set-up assumes that communication is reliable and components do not fail.

*Load generation.* Load on the system is induced by the master when it creates slave processes and allocates *tasks*. The total number of slaves in one run can be set via the parameter $n$. Tasks are allocated to slave processes by the master, and consist of one or more *work requests* that a slave receives, handles, and relays back. A slave terminates its execution when all of its allocated work requests have been processed *and* acknowledged by the master. The number of work requests that *can* be batched in a task is controlled by the parameter $w$; the *actual* batch size per slave is then drawn randomly from a normal distribution with mean $\mu = w$ and standard deviation $\sigma = \mu \times 0.02$. This induces a degree of variability in the amount of work requests exchanged between master and slaves. The master and slaves communicate *asynchronously*: an allocated work request is delivered to a slave process' incoming work queue where it is eventually handled. Work responses issued by a slave are queued and processed similarly on the master.

*Load configuration.* We consider *three load profiles* (see fig. 3 for examples) that determine how the creation of slaves is distributed along the load timeline $t$. The timeline is modelled as a sequence of *discrete logical time units* representing instants at which a new set of slaves is created by the master. *Steady* loads replicate executions where a system operates under stable conditions. These are modelled on a homogeneous Poisson distribution with *rate* $\lambda$, specifying the mean number of slaves that are created at each time instant along the load timeline with duration $t = \lceil n/\lambda \rceil$. *Pulse* loads emulate settings where a system experiences gradually increasing load peaks. The Pulse load shape is parametrised by $t$ and the *spread*, $s$, that controls how slowly or sharply the system load increases as it approaches its maximum peak, halfway along $t$. Pulses are modelled on a normal distribution with $\mu = t/2$ and $\sigma = s$. *Burst* loads capture scenarios where a system is stressed due to load spikes; these are based on a log-normal distribution with $\mu = \ln(m^2/\sqrt{p^2+m^2})$ and $\sigma = \sqrt{ln(1+p^2/m^2)}$, where $m = t/2$, and parameter $p$ is the *pinch* controlling the concentration of the initial load burst.

*Wall-clock time.* A load profile created for a logical timeline $t$ is put into effect by the master process when the system starts running. The master *does not* create the slave processes that are set to execute in a particular time unit *in one go*, since this naïve strategy risks saturating the system, deceivingly increasing the load. In doing so, the system may become overloaded not because the mean request rate is high, but because the created slaves overwhelm the master when they send their requests all at once. We address this issue by introducing the notion of *concrete time* that maps one discrete time unit in $t$ to a real time *period*, $\pi$. The parameter $\pi$ is given in milliseconds (ms), and defaults to 1000 ms.

*Slave scheduling.* The master process employs a scheduling scheme to distribute the creation of slaves uniformly across the time period $\pi$. It makes use of three queues: the *Order* queue, *Ready* queue, and *Await* queue, denoted by $Q_{\mathrm{O}}$, $Q_{\mathrm{R}}$, and $Q_{\mathrm{A}}$ respectively. $Q_{\mathrm{O}}$ is initially populated with the load profile, step ① in fig. 1a. The load profile consists of an array with $t$ elements—each corresponding to a discrete time instant in $t$—where the value $l$ of every element indicates the number of slaves to be created at that instant. Slaves, $S_1, S_2, \ldots, S_n$, are scheduled and created in *rounds*, as follows. The master picks the first element from $Q_{\mathrm{O}}$

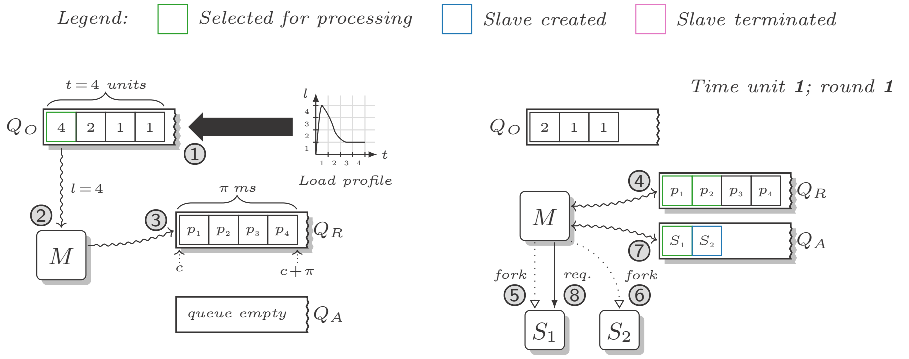

(a) Master schedules the first batch of four slaves for execution in $Q_{\mathrm{R}}$

(b) Slaves $S_1$ and $S_2$ created and added to $Q_{\mathrm{A}}$; a work request is sent to $S_1$

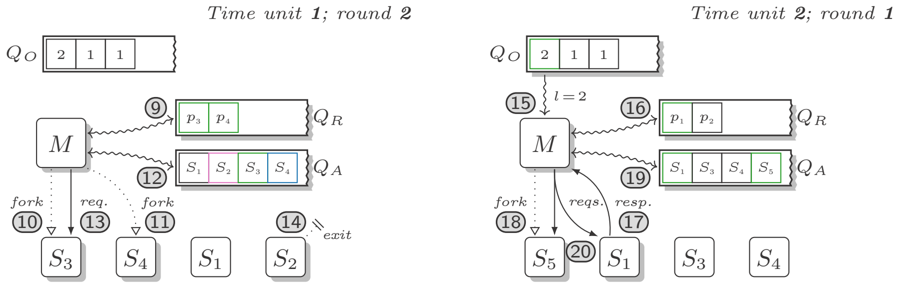

(c) Slaves $S_3$ and $S_4$ created and added to $Q_{\mathrm{A}}$; slave $S_2$ completes its execution

(d) $Q_{\mathrm{R}}$ becomes empty; master schedules the next batch of two slaves

Fig. 1: Master $M$ scheduling slave processes $S_j$ and allocating work requests to compute the upcoming schedule, step ②, that starts at the *current* time, $c$, and finishes at $c+\pi$. A series of $l$ time points, $p_1, p_2, \ldots, p_l$, in the schedule period $\pi$ are *cumulatively* calculated by drawing the next $p_i$ from a normal distribution with $\mu = \lceil \pi/l \rceil$ and $\sigma = \mu \times 0.1$. Each time point stipulates a moment in *wall-clock* time when a new slave $S_j$ is to be created; this set of time points is *monotonic*, and constitutes the Ready queue, $Q_{\mathrm{R}}$, step ③. The master checks $Q_{\mathrm{R}}$, step ④ in fig. 1b, and creates the slaves whose time point $p_i$ is smaller than or equal to the current wall-clock time[4], steps ⑤ and ⑥ in fig. 1b. The time point $p_i$ of a newly-created slave is removed from $Q_{\mathrm{O}}$, and an entry for the corresponding slave $S_j$ is appended to the Await queue $Q_{\mathrm{A}}$; this is shown in step ⑦ for $S_1$ and $S_2$. Slaves in $Q_{\mathrm{A}}$ are now ready to receive work requests from the master process, *e.g.* step ⑧. $Q_{\mathrm{A}}$ is traversed by the master at this stage so that work requests can be allocated to existing slaves. The master continues processing queue $Q_{\mathrm{R}}$ in subsequent rounds, creating slaves, issuing work requests, and updating $Q_{\mathrm{R}}$ and $Q_{\mathrm{A}}$ accordingly as shown in steps ⑨–⑬

[4] We assume that the platform scheduling the master and slave processes is *fair*.

in fig. 1c. At any point, the master can receive responses, *e.g.* step (17) in fig. 1d; these are *buffered* inside the masters' incoming work queue and handled once the scheduling and work allocation phases are complete. A *fresh* batch of slaves from $Q_{\mathrm{O}}$ is scheduled by the master whenever $Q_{\mathrm{R}}$ becomes empty, step (15), and the described procedure is repeated. The master stops scheduling slaves when all the entries in $Q_{\mathrm{O}}$ are processed. It then transitions to *work-only* mode, where it continues allocating work requests and handling incoming responses from slaves.

*Reactiveness and task allocation.* Systems generally respond to load with differing rates, due to the computational complexity of the task at hand, IO, or slowdown when the system itself becomes gradually loaded. We simulate these phenomena using the parameters $\mathrm{Pr}(send)$ and $\mathrm{Pr}(recv)$. The master *interleaves* the processing of work requests to allocate them uniformly among the various slaves: $\mathrm{Pr}(send)$ and $\mathrm{Pr}(recv)$ bias this behaviour. Specifically, $\mathrm{Pr}(send)$ controls the probability that a work request is sent by the master to a slave, whereas $\mathrm{Pr}(recv)$ determines the probability that a work response received by the master is processed. Sending and receiving is *turn-based* and modelled on a Bernoulli trial. The master picks a slave $S_j$ from $Q_{\mathrm{A}}$ and sends *at least* one work request when $X \leq \mathrm{Pr}(send)$, *i.e.,* the Bernoulli trial succeeds; $X$ is drawn from a uniform distribution on the interval [0,1]. Further requests to the *same* slave are allocated following this scheme (steps (8), (13) and (20) in fig. 1) and the entry for $S_j$ in $Q_{\mathrm{A}}$ is updated accordingly with the number of work requests remaining. When $X > \mathrm{Pr}(send)$, *i.e.,* the Bernoulli trial fails, the slave misses its turn, and the next slave in $Q_{\mathrm{A}}$ is picked. The master also queries its incoming work queue to determine whether a response can be processed. It dequeues one response when $X \leq \mathrm{Pr}(recv)$, and the attempt is repeated for the next response in the queue until $X > \mathrm{Pr}(recv)$. The master signals slaves to terminate once it acknowledges all of their work responses (*e.g.* step (14)). Due to the load imbalance that may occur when the master becomes overloaded with work responses relayed by slaves, dequeuing is repeated $|Q_{\mathrm{A}}|$ times. This encourages an even load distribution in the system as the number of slaves *fluctuates* at runtime.

#### 2.2 Realisability

The set-up detailed in sec. 2.1 is easily translatable to the actor model of computation [2]. In this model, the basic units of decomposition are *actors*: concurrent entities that do not share mutable memory with other actors. Instead, they interact via *asynchronous messaging*. Each actor owns an incoming message buffer called the *mailbox*. Besides sending and receiving messages, an actor can also *fork* other child actors. Actors are uniquely addressable via a dynamically-assigned *identifier*, often referred to as the PID. Actor frameworks such as Erlang [16], Akka [55] for Scala [51], and Thespian [53] for Python [44] implement actors as *lightweight* processes to enable highly-scalable architectures that span multiple machines. The terms *actor* and *process* are used interchangeably henceforth.

*Implementation.* We use Erlang to implement the set-up of sec. 2.1. Our implementation maps the master and slave processes to actors, where slaves are forked by the master via the Erlang function `spawn()`; in Akka and Thespian `ActorContext.spawn()` and `Actor.createActor()` can be respectively used to the same effect. The work request queues for both master and slave processes coincide with actor mailboxes. We abstract the task computation and model work requests as Erlang messages. Slaves emulate no delay, but respond instantly to work requests once these have been processed; delay in the system can be induced via parameters $\Pr(send)$ and $\Pr(recv)$. To maximise efficiency, the Order, Ready and Await queues used by our scheduling scheme are maintained *locally* within the master. The master process keeps track of other details, such as the total number of work requests sent and received, to determine when the system should stop executing. We extend the parameters in sec. 2.1 with a *seed* parameter, $r$, to fix the Erlang pseudorandom number generator to output reproducible number sequences.

#### 2.3 Measurement Collection

To give a multi-faceted view of runtime overhead, we extend the approach in [8] and, apart from the *(i)* mean *execution duration*, measured in seconds (s), we also collect the *(ii)* mean *scheduler utilisation*, as a percentage of the total available capacity, *(iii)* mean *memory consumption*, measured in GB, and, *(iv)* mean *response time (RT)*, measured in milliseconds (ms). Our definition of runtime overhead encompasses all four metrics. Measurement taking largely depends on the platform on which the benchmark executes, and one often leverages *platform-specific* optimised functionality in order to attain high levels of efficiency. Our implementation relies on the functionality provided by the Erlang ecosystem.

*Sampling.* We collect measurements centrally using a special process, called the *Collector*, that samples the runtime to obtain periodic snapshots of the execution environment (see fig. 2). Sampling is often necessary to induce low overhead in the system, especially in scenarios where the system components are sensitive to latency [32]. Our sampling frequency is set to 500 ms: this figure was determined empirically, whereby the measurements gathered are neither too coarse, nor excessively fine-grained such that sampling affects the runtime. Every sampling snapshot combines the four metrics mentioned above and formats them as records that are written *asynchronously* to disk to minimise IO delays.

*Performance metrics.* Memory and scheduler readings are gathered via the Erlang Virtual Machine (EVM). We sample scheduler—rather than CPU utilisation at the OS-level—since the EVM keeps scheduler threads momentarily spinning to remain reactive; this would inflate the metric reading. The overall system responsiveness is captured by the mean RT metric. Our Collector exposes a hook that the master uses to obtain *unique timestamps*, step ① in fig. 2. These are embedded in all work request messages the master issues to slaves. Each timestamp enables the Collector to track the time taken for a message to travel from the master to a slave and back, *including* the time it spends in the master's mailbox until dequeued, *i.e.,* the round-trip in steps ②–⑤. To efficiently compute the RT, the Collector samples the total number of messages exchanged between the master and slaves, and calculates the mean using Welford's online algorithm [62].

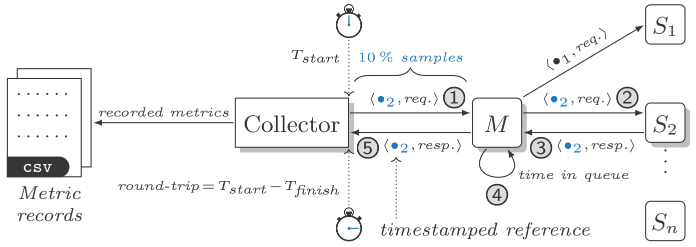

Fig. 2: Collector tracking the round-trip time for work requests and responses

## 3 Evaluation

We evaluate our synthetic benchmarking tool developed as described in Sec. 2 in a number of ways. In sec. 3.1, we discuss sanity checks for its measurement collection mechanisms, and assess the repeatability of the results obtained from the synthetic system executions. Crucially, sec. 3.1 provides evidence that the benchmarking tool is sufficiently expressive to cover a number of execution profiles that are shown to emulate realistic scenarios. Sec. 3.2 demonstrates the utility of the features offered by our tool for the purposes of assessing RV tools.

*Experiment set-up.* We define an *experiment* to consist of ten benchmarks, each performed by running the system set-up with incremental loads. Our experiments were performed on an Intel Core i7 M620 64-bit machine with 8GB of memory, running Ubuntu 18.04 LTS and Erlang/OTP 22.2.1.

### 3.1 Benchmark Expressiveness and Veracity

The parameters for the tool detailed in sec. 2.1 can be configured to model a range of master-slave scenarios. However, not all of these configurations are meaningful in practice. For example, setting $\Pr(send) = 0$ does not enable the master to allocate work requests to slaves; with $\Pr(send) = 1$, this allocation is enacted sequentially, defeating the purpose of a concurrent master-slave system. In this section, we establish a set of parameter values that model experiment set-ups whose behaviour *approximates* that of master-slave systems typically found in practice. Our experiments are conducted with $n$=500k slaves and $w$=100 work requests per slave. This generates $\approx n \times w \times$(work requests and responses)=100M message exchanges between the master and slaves. We initially fix $\Pr(send) = \Pr(recv) = 0.9$, and choose a Steady (*i.e.,* Poisson process) load profile since this features in industry-strength load testing tools such as Tsung [49] and JMeter [3]. Fig. 3 shows the load applied at each benchmark run, *e.g.* on the tenth run, the benchmark uses $\approx$ 5k slaves/s. The total loading time is set to $t = 100$s.

*Measurement precision.* A series of trials were conducted to select the appropriate sampling window size for the RT. This step is crucial because it directly affects the capability of the benchmark to scale in terms of its number of slave processes and work requests. Our RT sampling of sec. 2.3 (see also fig. 2) was calibrated by taking various window sizes over numerous runs for different load profiles of $\approx 1$M slaves. The results were compared to the *actual* mean calculated on *all* work request and response messages exchanged between master and slaves. Window sizes close to 10 % yielded the best results ($\approx \pm 1.4\%$ discrepancy from the actual RT). Smaller window sizes produced excessive discrepancy; larger sizes induced noticeably higher system loads. We also cross-checked the precision of our sampling method of the scheduler utilisation against readings obtained via the Erlang Observer tool [16] to confirm that these coincide.

*Experiment repeatability.* Data variability affects the *repeatability* of experiments. It also plays a role when determining the number of repeated readings, $k$, required before the data measured is deemed *sufficiently representative.* Choosing the lowest $k$ is crucial when experiment runs are time consuming. The *coefficient of variation* (CV)—*i.e.,* the ratio of the standard deviation to the mean, $\text{CV} = \frac{\sigma}{\bar{x}} \times 100$—can be used to establish the value of $k$ empirically, as follows. Initially, the $\text{CV}_k$ for one batch of experiments for some number of repetitions $k$ is calculated. The result is then compared to the $\text{CV}_{k'}$ for the next batch of repetitions $k' = k + b$, where $b$ is the step size. When the difference between successive CV metrics $k'$ and $k$ is sufficiently small (for some percentage $\epsilon$), the value of $k$ is chosen, otherwise the described procedure is repeated with $k'$. Crucially, this condition must hold for *all variables* measured in the experiment before $k$ can be fixed. For the results presented next, the CV values were calculated manually. The mechanism that determines the CV automatically is left for future work.

*Data variability.* The data variability between experiments can be reduced by seeding the Erlang pseudorandom number generator (parameter $r$ in sec. 2.2) with a constant value. This, in turn, tends to require fewer repeated runs before the metrics of interest—scheduler utilisation, memory consumption, RT, and execution duration—converge to an acceptable CV. We conduct experiment sets with three, six and nine repetitions. For the majority of cases, the CV for our metrics is *lower* when a fixed seed is used, by comparison to its unseeded counterpart. In fact, very low CV values for the scheduler utilisation, memory consumption, RT, and execution duration, 0.17 %, 0.15 %, 0.52 % and 0.47 % respectively, were obtained with three repeated runs. We thus set the number of repetitions to *three* for *all* experiment runs in the sequel. Note that fixing the seed *still* permits the system to exhibit a modicum of variability that stems from the inherent *interleaved execution* of components due to process scheduling.

*Load profiles.* Our tool is expressive enough to generate the load profiles introduced in sec. 2.1 (see fig. 3), enabling us to gauge the behaviour of monitoring set-ups under varying forms of loads. These loads make it possible to mock specific system scenarios that test different implementation aspects. For example, a benchmark configured with load surges could uncover buffer overflows in a particular monitoring implementation that only arise under stress when the length of the request queue exceeds some preset length.

*System reactivity.* The reactivity of the master-slave system correlates with the idle time of each slave which, in turn, affects the capacity of the system to *absorb*

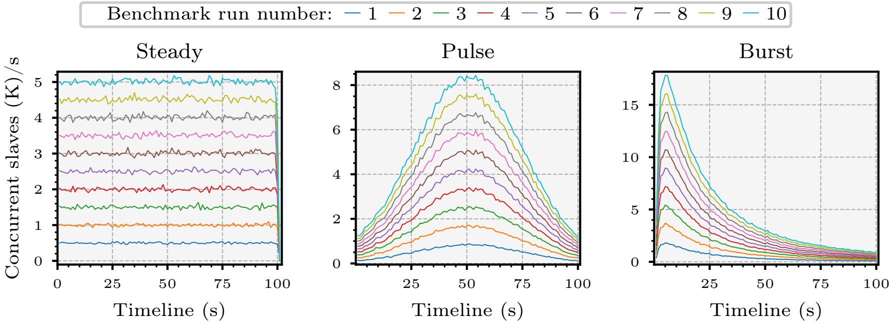

Fig. 3: Steady, Pulse and Burst load distributions of 500 k slaves for 100 s overheads. Since this can skew the results obtained when assessing overheads, it is imperative that the benchmarking tool provides methods to control this aspect. The parameters $\Pr(send)$ and $\Pr(recv)$ regulate the speed with which the system reacts to load. We study how these parameters affect the overall performance of system models set up with $\Pr(send) = \Pr(recv) \in \{0.1, 0.5, 0.9\}$. The results are shown in fig. 4, where each metric (*e.g.* memory consumption) is plotted against the total number of slaves. At $\Pr(send)$=$\Pr(recv)$=0.1, the system has the lowest RT out of the three configurations (bottom left), as indicated by the gentle linear increase of the plot. One may expect the RT to be *lower* for the system models configured with probability values of 0.5 and 0.9. However, we recall that with $\Pr(send) = 0.1$, work requests are allocated infrequently by the master, so that slaves are *often idle*, and can *readily* respond to (low numbers of) incoming work requests. At the same time, this prolongs the execution duration, when compared to that of the system set with $\Pr(send) = \Pr(recv) \in \{0.5, 0.9\}$ (bottom right). This effect of slave idling can be gleaned from the relatively lower scheduler utilisation as well (top left). Idling increases memory consumption (top right), since slaves created by the master typically remain alive for extended periods. By contrast, the plots set with $\Pr(send) = \Pr(recv) \in \{0.5, 0.9\}$ exhibit markedly gentler gradients in the memory consumption and execution duration charts; corresponding linear slopes can be observed in the RT chart. This indicates that values between 0.5 and 0.9 yield system models that: *(i)* consume reasonable amounts of memory, *(ii)* execute in respectable amounts of time, and *(iii)* maintain tolerable RT. Since master-slave architectures are typically employed in settings where high throughput is demanded, choosing values smaller than 0.5 goes against this principle. In what follows, we opt for $\Pr(send) = \Pr(recv) = 0.9$.

*Emulation veracity.* Our benchmarks can be configured to closely model *realistic* web server traffic where the request intervals observed at the server are known to follow a Poisson process [31,43,37]. The probability distribution of the RT of web application requests is generally right-skewed, and approximates log-normal [31,20] or Erlang distributions [37]. We conduct three experiments using *Steady loads* fixed with $n = 10$k for $\Pr(send) = \Pr(recv) \in \{0.1, 0.5, 0.9\}$ to

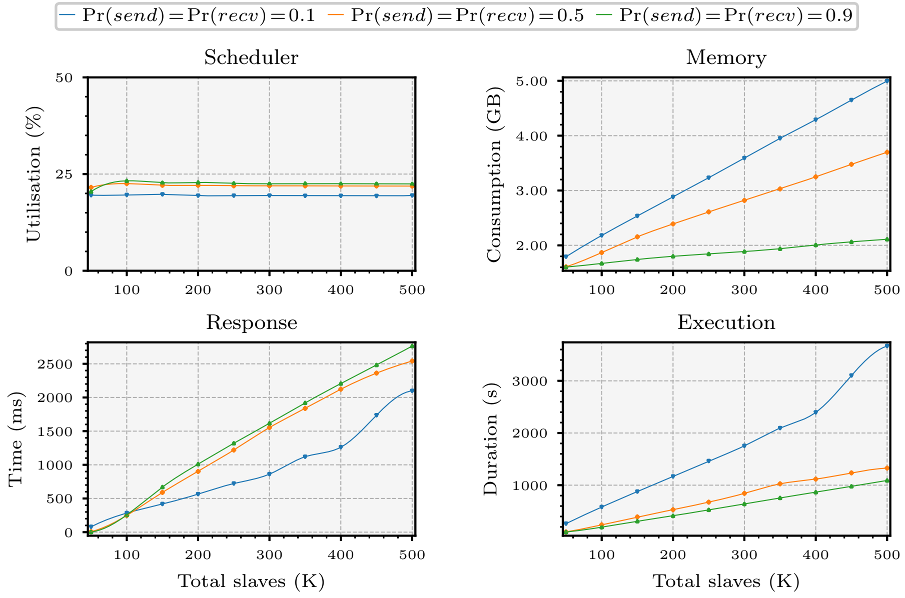

Fig. 4: Performance benchmarks of system models for Pr(*send*) and Pr(*recv*)

establish whether the RT in our system set-ups resembles the aforementioned distributions. Our results, summarised in fig. 5, were obtained by estimating the parameters for a set of candidate probability distributions (*e.g.* normal, log-normal, gamma, *etc.*) using maximum likelihood estimation [56] on the RT obtained from *each* experiment. We then performed goodness-of-fit tests on these parametrised distributions using the Kolmogorov-Smirnov test, selecting the most appropriate RT fit for each of the three experiments. The fitted distributions in fig. 5 indicate that the RT of our system models follows the findings reported in [31,20,37]. This makes a strong case in favour of our benchmarking tool striking a balance between the *realism* of benchmarks based on OTS programs and the *controllability* offered by synthetic benchmarking. Lastly, we point out that fig. 5 matches the observations made in fig. 4, which show an increase in the mean RT as the system becomes more reactive. This is evident in the histogram peaks that grow shorter as Pr(*send*) = Pr(*recv*) progresses from 0.1 to 0.9.

#### 3.2 Case Study

We demonstrate how our benchmarking tool can be used to assess the runtime overhead comprehensively via a concurrent RV case study. By controlling the benchmark parameters and subjecting the system to specific workloads, we show that our multi-faceted view of overhead reveals nuances in the observed runtime behaviour, benefitting the interpretation of empirical results. We further assess the veracity of these synthetic benchmarks against the overhead measured from a use case that considers industry-strength OTS applications.

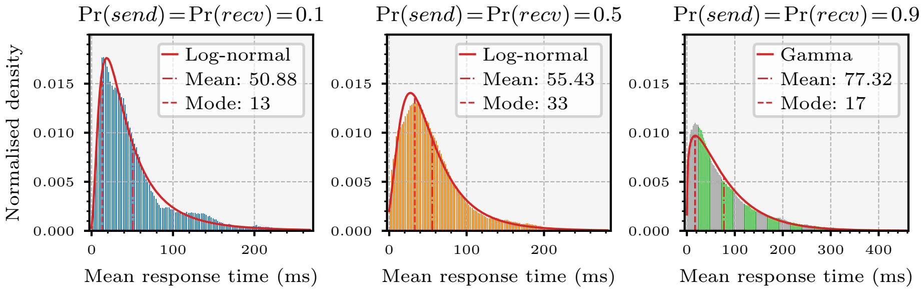

Fig. 5: Fitted probability distributions on RT for Steady loads for $n = 10$k

**The RV Tool** We use a RV tool to objectively compare the conclusions derived from our synthetic benchmarks against those obtained from the experiment set up with the OTS applications. The tool under scrutiny targets concurrent Erlang programs [4]. It synthesises *automata-like* monitors from sHML specifications [26] and *inlines* them into the system via *code injection* by manipulating the program abstract syntax tree. Inline instrumentation underlies various other state-of-the-art RV tools, such as JavaMOP [36], MarQ [54], Java-MaC [38] and RiTHM [47]. sHML is a fragment of the Hennessy-Milner Logic with recursion [41] that can express all regular safety properties [26]. The tool augments it to handle pattern matching and data dependencies for three kinds of event patterns, namely *send* and *receive* actions, denoted by ! and ? respectively, and process *crash*, denoted by $\star$. This suffices to specify properties of both the master and slave processes, resulting in the set-up depicted in fig. 6a. For instance, the recursive property $\varphi_s$ describes an *invariant* of the master-slave communication protocol (from the slave's point of view), stating that '*a slave processing integer successor requests should not crash*':

$$\mathsf{max}\,X.\Big(\overbrace{[\backslash Slv\,\star]\,\mathsf{ff}}^{①}\wedge\underbrace{\overbrace{[\backslash Slv\,?\,\backslash Req]}^{2.1}\big(\overbrace{\overbrace{[Slv\,\star]\,\mathsf{ff}}^{3.1}\wedge\overbrace{[Slv\,!\,(Req+1)]\,X}^{3.2}}^{③}\big)}_{②}\Big) \qquad (\varphi_s)$$

The key construct in sHML is the modal formula $[p]\varphi$, stating that *whenever* a satisfying system exhibits an event $e$ matching pattern $p$, its continuation then satisfies $\varphi$. In property $\varphi_s$, the invariant—denoted by recursion binder $\mathsf{max}X$—asserts that a slave *Slv* does not crash, specified by sub-formula ①. It further stipulates in sub-formula ② that when a request-carrying payload, *Req* is received, (2.1), *Slv* cannot crash, (3.1), *and* if the slave replies to *Req* with the payload *Req*+1, the property *recurses* on variable $X$, (3.2). Action patterns use two types of value variables: binders, $\backslash x$, that are pattern-matched to concrete values learnt at runtime, and variable instances, $x$, that are bound by the respective binders and instantiated to concrete data via pattern matching at runtime. This induces the usual notion of free and bound value variables; we assume closed terms. For example, when checking property $\varphi_s$ against the trace event `pid?42`, the analysis unfolds the sub-formula guarded by $\mathsf{max}X$, matching the event with the pattern $\backslash Slv?\backslash Req$ in (2.1). Variables *Slv* and *Req* are substituted with `pid` and `42` respectively in property $\varphi_s$, leaving the residual formula:

$$[\mathtt{pid}\star]\mathsf{ff}\wedge[\mathtt{pid}!(42+1)]\mathsf{max}\,X.\begin{pmatrix}[\backslash Slv\star]\mathsf{ff}\wedge\\ [\backslash Slv?\backslash Req]\big([Slv\star]\mathsf{ff}\wedge[Slv!(Req+1)]X\big)\end{pmatrix}$$

The RV tool under scrutiny produces inlined monitor code that executes in the same process space of system components (see fig. 6a), yielding the lowest possible amount of runtime overhead. This enables us to scale our benchmarks to considerably high loads. Our experiments focus on correctness properties that are *parametric* w.r.t. to system components [7,19,54,48]: with this approach, monitors need not interact with one another and can reach verdicts independently. Verdicts are communicated by monitors to a central entity that records the expected number of verdicts in order to determine when the experiment can be stopped. The set of properties used in our benchmarks translate to monitors that loop continually to exert the maximum level of runtime overhead possible.

Fig. 6b shows the monitor synthesised from property $\varphi_s$, consisting of states $Q_0$, $Q_1$, the rejection state ✗, and inconclusive state ?. The rejection state corresponds to a *violation* of the property, *i.e.*, $\mathsf{ff}$, whereas the *inconclusive* state is reached when the analysed trace events do not contain enough information to enable the monitor to transition to any other state. Both of these states are sinks, modelling the irrevocability of verdicts [24,26]. The modality $[\backslash Slv?\backslash Req]$ in property $\varphi_s$ corresponds to the transition between $Q_0$ and $Q_1$ in fig. 6b. The monitor follows this transition when it analyses the trace event $\mathtt{pid_1?d_1}$ exhibited by the slave with PID $\mathtt{pid_1}$ when it receives data payload $\mathtt{d_1}$ from the master; as a side effect, the transition binds the variable *Slv* to $\mathtt{pid_1}$ and *Req* to $\mathtt{d_1}$ in

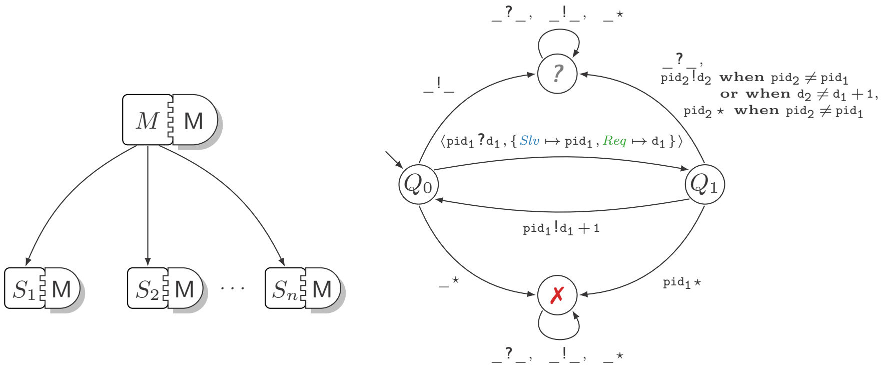

(a) Inlined runtime monitors (b) Synthesised monitor from property $\varphi_s$

Fig. 6: Synthesised monitors instrumented with master and slave processes state $Q_1$. From $Q_1$, the monitor transitions to $Q_0$ only when the event $\mathtt{pid_1!d_2}$ is analysed, where $\mathtt{d_2} = \mathtt{d_1} + 1$ and $\mathtt{pid_1}$ is the slave PID (previously) bound to *Slv*. From $Q_0$ and $Q_1$, the rejection state ✗ can be reached when a crash event is analysed. In the case of $Q_0$, the transition to ✗ is followed for *any* crash event $\_\star$ (the wildcard _ denotes the *anonymous* variable). By contrast, the monitor reaches ✗ from $Q_1$ *only* when the slave with PID $\mathtt{pid_1}$ crashes, otherwise it transitions to the inconclusive state ?. Other transitions from $Q_0$ and $Q_1$ leading to ? follow a similar reasoning. Interested readers are encouraged to consult [25,6,5] for more information on the specification logic and monitor synthesis.

**Synthetic Benchmarks** We set the total number of slaves to $n = 20\text{k}$ for *moderate* loads and $n = 500\text{k}$ for *high* loads; $\Pr(send) = \Pr(recv)$ is fixed at 0.9 as in sec. 3.1. These configurations generate $\approx n \times w \times (\text{work requests and responses}) =$ 4M and 100M messages respectively to produce 8M and 200M analysable trace events per run. The pseudorandom number generator is seeded with a constant value and three experiment repetitions are performed for the Steady, Pulse and Burst load profiles (see fig. 3). A loading time of $t = 100\text{s}$ is used. Our results are summarised in figs. 7 and 8. Each chart in these figures plots the particular performance metric (*e.g.* memory consumption) for the system without monitors, *i.e.,* the *baseline*, together with the overhead induced by the RV monitors.

*Moderate loads.* Fig. 7 shows the plots for the system set with $n = 20\text{k}$. These loads are similar to those employed by the state-of-the-art frameworks to evaluate component-based runtime monitoring, *e.g.* [57,7,10,23,48] (ours are slightly higher). We remark that none of the benchmarks used in these works consider different load profiles: they either model load on a Poisson process, or fail to specify the kind of load used. In fig. 7, the execution duration chart (bottom right) shows that, regardless of the load profile used, the running time of each experiment is comparable to the baseline. With the moderate size of 20k slaves, the execution duration on its own does not give a detailed enough view of runtime overhead, despite the fact that our benchmarks provide a broad coverage in terms of the Steady, Pulse and Burst load profiles. This trend is mirrored in the scheduler utilisation plot (top left), where both baseline and monitored system induce a constant load of $\approx 17.5\%$. On this account, we deem these results to be *inconclusive.* By contrast, our three load profiles induce different overhead for the RT (bottom left), and, to a lesser extent, the memory consumption plots (top right). Specifically, when the system is subjected to a Burst load, it exhibits a surge in the RT for the baseline and monitored system alike, at $\approx 16\text{k}$ slaves. While this is not reflected in the consumption of memory, the Burst plots do exhibit a larger—albeit linear—rate of increase in memory when compared to their Steady and Pulse counterparts. The latter two plots once again show analogous trends, indicating that both Steady and Pulse loads exact similar memory requirements and exhibit comparable responsiveness under the respectable load of 20k slaves. Crucially, the data plots in fig. 7 *do not* enable us to confidently extrapolate our results. The edge case in the RT chart for Burst plots raises the question of whether the surge in the trend observed at $\approx 16\text{k}$ remains consistent

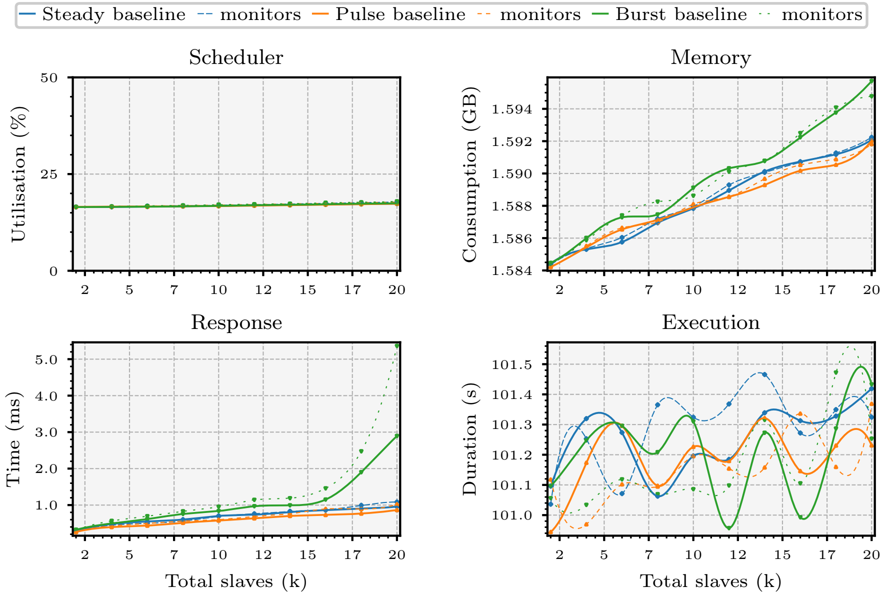

Fig. 7: Mean runtime overhead for master and slave processes (20 k slaves)

when the number of slaves goes beyond 20k. Similarly, although for a different reason, the execution duration plots do not allow us to distinguish between the overhead induced by monitors for different loads on this small scale—this occurs due to the *perturbations* introduced by the underlying OS (*e.g.* scheduling other processes, IO, *etc.*) that affect the sensitive time keeping of benchmarks.

*High loads.* We increase the load to $n = 500$k slaves to determine whether our benchmark set-up can adequately scale, and show how the monitored system performs under stress. The RT chart in fig. 8 indicates that for Burst loads (bottom left), the overhead induced by monitors *grows linearly* in the number of slaves. This contradicts the results in fig. 7, confirming our supposition that moderate loads may provide scant empirical evidence to extrapolate to general conclusions. However, the memory consumption for Burst loads (top right) exhibits similar trends to the ones in fig. 7. Subjecting the system to high loads renders discernible the discrepancy between the RT and memory consumption gradients for the Steady and Pulse plots that appeared to be similar under the moderate loads of 20k slaves. Considering the execution duration chart (bottom right of fig. 8) as the *sole* indicator of overhead could *deceivingly suggest* that runtime monitoring induces virtually identical overhead for the distinct load profiles of fig. 3. However, this erroneous observation is easily refuted by the memory consumption and RT plots that show otherwise. This stresses the merit of gathering multi-faceted metrics to assist in the interpretation of runtime overhead.

We extend the argument for multi-faceted views to the scheduler utilisation metric in fig. 8 that reveals a subtle aspect of our concurrent set-up. Specifically,

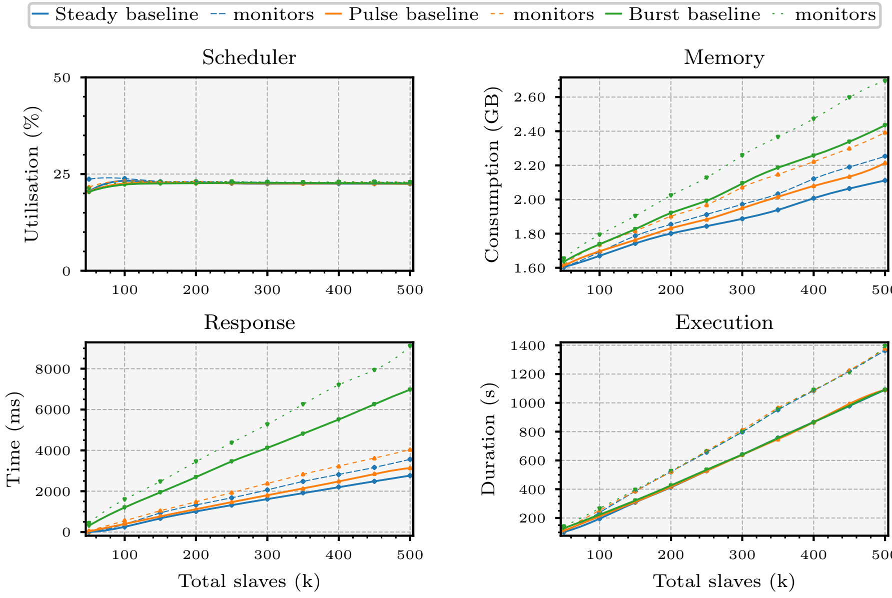

Fig. 8: Mean runtime overhead for master and slave processes (500 k slaves)

the charts show that while the execution duration, RT and memory consumption plots grow in the number of slave processes, scheduler utilisation stabilises at ≈ 22.7%. This is partly caused by the master-slave design that becomes susceptible to bottlenecks when the master is overloaded with requests [61]. In addition, the preemptive scheduling of the EVM [16] ensures that the master *shares* the computational resources of the same machine with the rest of the slaves. We conjecture that, in a distributed set-up where the master resides on a *dedicated* node, the overall system throughput may be further pushed. Fig. 8 also attests to the utility of having a benchmarking framework that scales considerably well to increase the chances of detecting potential trends. For instance, the evidence gathered earlier in fig. 7 could have misled one to assert that the RV tool under scrutiny scales poorly under Burst loads of moderate and larger sizes.

**An OTS Application Use Case** We evaluate the overheads induced by the RV tool under scrutiny using a third-party industry-strength web server called Cowboy [33], and show that the conclusions we draw are *in line* with those reported earlier for our synthetic benchmark results. Cowboy is written in Erlang and built on top of Ranch [34]—a socket acceptor pool for TCP protocols that can be used to develop custom network applications. Cowboy relies on Ranch to manage its socket connections, but delegates HTTP client requests to *protocol handlers* that are forked dynamically by the web server to handle each request independently. This architecture follows closely our master-slave set-up of sec. 2.1 which abstracts details such as TCP connection management and

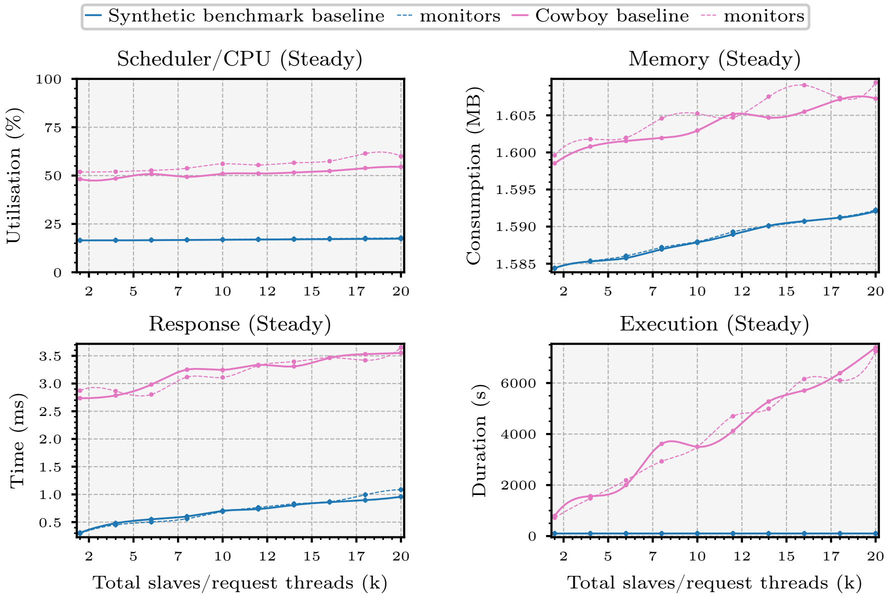

Fig. 9: Mean overhead for synthetic and Cowboy benchmarks (20 k threads)

HTTP protocol parsing. We generate load on Cowboy using the popular stress testing tool JMeter [3] to issue HTTP requests from a dedicated machine residing on the same network where Cowboy is hosted. The latter machine is the one used in the experiments discussed earlier. To emulate the typical behaviour of web clients (*e.g.* browsers) that fetch resources via multiple HTTP requests, our Cowboy application serves files of various sizes that are randomly accessed by JMeter during the benchmark. In our experiments, we monitored fragments of the Cowboy and Ranch communication protocol used to handle client requests.

*Moderate loads.* Fig. 9 plots our results for *Steady* loads from fig. 7, together with the ones obtained from the Cowboy benchmarks; JMeter did not enable us to reproduce the Pulse and Burst load profiles. For our Cowboy benchmarks, we fixed the total number of JMeter request threads to 20k over the span of 100s, where each thread issued 100 HTTP requests. This configuration coincides with parameter settings used in the experiments of fig. 7. In fig. 9, the scheduler utilisation, memory consumption and RT charts (top, bottom left) show a correspondence between the baseline plots of our synthetic benchmarks and those taken with Cowboy and JMeter. This indicates that, for these metrics, our synthetic system model exhibits *analogous characteristics* to the ones of the OTS system, under the chosen load profile. The argument can be extended to the monitored versions of these systems which follow identical trends. We point out the similarity in the RT trends of our synthetic and Cowboy benchmarks, despite the fact that the latter set of experiments were conducted over a local network. This suggests that, for our single-machine configuration, the synthetic master-slave benchmarks manage to adequately capture local network conditions. The gaps separating the plots of the two experiment set-ups stem from the implementation specifics of Cowboy and our synthetic model. This discrepancy in measurements also depends on the method used to gather runtime metrics, *e.g.* JMeter cannot sample the EVM directly, and measures CPU as opposed to scheduler utilisation. The deviation in execution duration plots (bottom right) arises for the same reason.

*High loads.* Our efforts to run tests with 500k request threads where stymied by the scalability issues we experienced with Cowboy and JMeter on our set-up.

### 4 Conclusion

Concurrent RV necessitates benchmarking tools that can *scale dynamically* to accommodate considerable load sizes, and are able to provide a *multi-faceted view* of runtime overhead. This paper presents a benchmarking tool that fulfils these requirements. We demonstrate its implementability in Erlang, arguing that the design is easily instantiatable to other actor frameworks such as Akka and Thespian. Our set-up emulates various system models through configurable parameters, and scales to reveal behaviour that emerges only when software is pushed to its limit. The benchmark harness gathers different performance metrics, offering a multi-faceted view of runtime overhead that, to wit, other state-of-the-art tools do not currently offer. Our experiments demonstrate that these metrics benefit the interpretation of empirical measurements: they increase visibility that may spare one from drawing insufficiently general, or otherwise, erroneous conclusions. We establish that—despite its synthetic nature—our master-slave model faithfully approximates the mean response times observed in realistic web server traffic. We also compare the results of our synthetic benchmarks against those obtained from a real-world use case to confirm that our tool captures the behaviour of this realistic set-up. It is worth noting that, while our empirical measurements of secs. 3.1 and 3.2 depend on the implementation language, our conclusions are transferrable to other frameworks, *e.g.* Akka and Play [42].

*Related work.* There are other less popular benchmarks targeting the JVM besides those mentioned in sec. 1. Renaissance [52] employs workloads that leverage the concurrency primitives of the JVM, focussing on the performance of compiler optimisations similar to DaCapo and ScalaBench. These benchmarks gather metrics that measure software quality and complexity, as opposed to metrics that gauge runtime overhead. The CRV suite [8] aims to standardise the evaluation of RV tools, and mainly focusses on RV for monolithic programs. We are unaware of RV-centric benchmarks for concurrent systems such as ours. In [43], the authors propose a queueing model to analyse web server traffic, and develop a benchmarking tool to validate it. Their model coincides with our master-slave set-up, and considers loads based on a Poisson process. A study of message-passing communication on parallel computers conducted in [31] uses systems loaded with different numbers of processes; this is similar to our approach. Importantly, we were able to confirm the findings reported in [43] and [31] (sec. 3.1).
